# Methylation levels of the "*long interspersed nucleotide element-1" *repetitive sequences predict survival of melanoma patients

**DOI:** 10.1186/1479-5876-9-78

**Published:** 2011-05-26

**Authors:** Luca Sigalotti, Elisabetta Fratta, Ettore Bidoli, Alessia Covre, Giulia Parisi, Francesca Colizzi, Sandra Coral, Samuele Massarut, John M Kirkwood, Michele Maio

**Affiliations:** 1 Cancer Bioimmunotherapy Unit, Centro di Riferimento Oncologico, Istituto di Ricovero e Cura a Carattere Scientifico, Aviano, Italy; 2 Biostatistics and Epidemiology Unit, Centro di Riferimento Oncologico, Istituto di Ricovero e Cura a Carattere Scientifico, Aviano, Italy; 3 Breast Surgery Unit, Centro di Riferimento Oncologico, Istituto di Ricovero e Cura a Carattere Scientifico, Aviano, Italy; 4 University of Pittsburgh School of Medicine, Pittsburgh, Pennsylvania, USA; 5 Division of Medical Oncology and Immunotherapy, Department of Oncology, University Hospital of Siena, Istituto Toscano Tumori, Siena, Italy

## Abstract

**Background:**

The prognosis of cutaneous melanoma (CM) differs for patients with identical clinico-pathological stage, and no molecular markers discriminating the prognosis of stage III individuals have been established. Genome-wide alterations in DNA methylation are a common event in cancer. This study aimed to define the prognostic value of genomic DNA methylation levels in stage III CM patients.

**Methods:**

Overall level of genomic DNA methylation was measured using bisulfite pyrosequencing at three CpG sites (CpG1, CpG2, CpG3) of the *Long Interspersed Nucleotide Element-1 *(*LINE-1*) sequences in short-term CM cultures from 42 stage IIIC patients. The impact of *LINE-1 *methylation on overall survival (OS) was assessed using Cox regression and Kaplan-Meier analysis.

**Results:**

Hypomethylation (i.e., methylation below median) at CpG2 and CpG3 sites significantly associated with improved prognosis of CM, CpG3 showing the strongest association. Patients with hypomethylated CpG3 had increased OS (P = 0.01, log-rank = 6.39) by Kaplan-Meyer analysis. Median OS of patients with hypomethylated or hypermethylated CpG3 were 31.9 and 11.5 months, respectively. The 5 year OS for patients with hypomethylated CpG3 was 48% compared to 7% for patients with hypermethylated sequences. Among the variables examined by Cox regression analysis, *LINE-1 *methylation at CpG2 and CpG3 was the only predictor of OS (Hazard Ratio = 2.63, for hypermethylated CpG3; 95% Confidence Interval: 1.21-5.69; *P *= 0.01).

**Conclusion:**

*LINE-1 *methylation is identified as a molecular marker of prognosis for CM patients in stage IIIC. Evaluation of *LINE-1 *promises to represent a key tool for driving the most appropriate clinical management of stage III CM patients.

## Background

Cutaneous melanoma (CM) is a very aggressive neoplasm of growing incidence and mortality in industrialized countries, and the leading cause of skin cancer-related deaths worldwide [1]. Surgery, in early phases of disease has curative potential for patients; for advanced CM conventional therapies have failed to prolong survival [2]. At present, the best predictor of 5-year survival is the clinico-pathological stage of disease, which defines overall survival (OS) rates ranging from 95% to 7% for stage I to IV patients, respectively [3]. However, within the same clinico-pathological stage category, patients often behave radically differently, and the current lack of prognostic molecular markers impairs our ability to identify CM patients with highly aggressive as opposed to more indolent courses of disease [4].

In mammals, DNA methylation of cytosine at the 5C-position in the context of CpG dinucleotides represents a major epigenetic mechanism controlling gene expression, chromosome X inactivation, imprinting and repression of endogenous parasitic sequences (for review see [5]). Global genomic DNA hypomethylation (i.e., overall reduction of the 5-methylcytosine content) is a frequent molecular event in cancer and has been observed in neoplastic cells of different histotypes [6]. Genomic hypomethylation might contribute to cancer development and progression through various mechanisms including generation of chromosomal instability, reactivation of transposable elements, and loss of imprinting [5]. Substantial decreases in the 5-methylcytosine content in the genome mainly reflect the hypomethylation of repetitive genomic sequences. Among these, methylation levels of the *Long Interspersed Nucleotide Element-1 *(*LINE-1*) may represent a surrogate marker for the overall level of genomic DNA methylation [7]. Preliminary investigations of *LINE-1 *methylation in solid tumors have identified increasingly greater hypomethylation of these sequences with progression of gastric and prostatic cancer [8,9]. Furthermore, decreased methylation of *LINE-1 *correlated with higher FIGO stage and advanced tumor grade of ovarian cancer [10]. Of interest, a increased hypomethylation of *LINE-1 *elements has been associated with poorer prognosis in colon and ovarian cancers [10,11]; however, these studies did not investigate the role of *LINE-1 *methylation as a prognostic factor in patients at identical stages of disease.

Despite these promising initial data, to the best of our knowledge no studies have investigated the influence of the overall level of genomic DNA methylation on CM prognosis. Accordingly, we investigated whether the extent of methylation of the *LINE-1 *repetitive elements may account for the differing survival patterns of CM patients of identical clinico-pathological stage of disease. The study was conducted on a series of 42 consecutive stage IIIC CM patients for whom the autologous short-term cell cultures were available. The latter were analyzed early during *in vitro *passage, and utilized instead of tumor tissues to overcome possible alterations in the evaluation of levels of *LINE-1 *methylation due to the unavoidable presence of contaminating normal cells. Results demonstrated that *LINE-1 *hypomethylation identifies CM patients with a significantly better prognosis as compared to those with hypermethylated *LINE-1 *sequences. These findings demonstrate that evaluation of *LINE-1 *methylation levels may greatly help in guiding the daily clinical management of CM patients, and provide a strong rationale for the development of a large prospective validation study.

## Methods

### Patients and cell cultures

Short-term cell cultures were established from metastatic lesions removed surgically from consecutive CM patients referred to the National Cancer Institute of Aviano (Italy) for stage III surgery from 1991 to 2007, as previously described [12]. Informed consent was obtained from patients. Autologous tumor cell cultures were successfully established from 30% of patients. The micrometastatic nature of lymph-node tumor tissues from AJCC stage IIIA patients precluded their use for cell culture generation, while short-term CM cultures were available only from 12 stage IIIB patients, and were excluded from the statistical analyses. Thus, the planned studies were conducted on a total of 42 available short-term cultures, identified as having been generated from CM patients classified as AJCC stage IIIC, who received highly heterogeneous treatments for their disease, including chemotherapy with different agents, immunotherapy, and radiotherapy. Short-term CM cell cultures were grown in RPMI 1640 Medium (Biochrome AG, Berlin, Germany) supplemented with 20% heat-inactivated fetal calf serum (Biochrome AG) and 2 mM L-glutamine (Biochrome AG). Four independent cultures of normal human melanocytes were purchased from Invitrogen (Milan, Italy), Gentaur (Brussels, Belgium), Provitro (Berlin, Germany), and ScienCell (Carlsbad, CA, USA), and were maintained in M254 Medium supplemented with Human Melanocyte Growth Supplement (Invitrogen). To minimize alterations potentially arising with extended *in vitro *culturing, all cell cultures were utilized for molecular assays at the 6^th ^*ex vivo *passage. Normal human Peripheral Blood Mononuclear Cells (PBMC) were separated from heparinized blood of 8 healthy donors by Biocoll (Biochrome AG) density gradient centrifugation (400 × g for 30 min) and used for molecular assays.

### LINE-1 bisulfite pyrosequencing analysis

**Figure 1:**
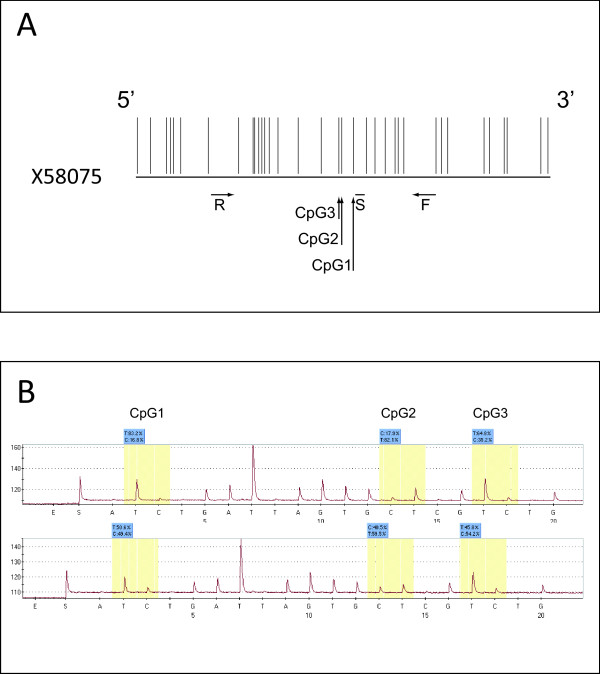
***LINE-1 *bisulfite pyrosequencing assay**. **A**. The region of the *LINE-1 *sequence [GenBank:X58075] utilized for the design of the assay is reported. Vertical bars indicate individual CpG sites. Horizontal lines indicate forward (F), reverse (R) and sequencing (S) primers. Vertical arrows indicate the CpG sites (CpG1, CpG2, CpG3) analyzed by pyrosequencing (adapted from [14]). **B**. Representative pyrograms for the methylation of *LINE-1 *repetitive sequences. Yellow shadowing highlights the 3 target regions (CpG1, 2, 3) in the pyrograms. T and C peaks indicate unmethylated and methylated cytosines, respectively. Accordingly, % of *LINE-1 *methylation at each site is defined by the % of the C base. Upper and lower panels are representative of short-term cultures of CM cells with low and high *LINE-1 *methylation, respectively.

Genomic DNA was extracted from short-term cultures of CM cells by proteinase K treatment followed by standard phenol/chloroform extraction and ethanol precipitation [13]. Bisulfite conversion was carried out on 500 ng genomic DNA using EZ DNA Methylation-Gold™ Kit (Zymo Research, Orange, CA, USA), according to the manufacturer's protocol. Methylation analysis of the *LINE-1 *elements was performed as previously described [7], with minor modifications. *LINE-1 *elements were amplified using 50 pmol each of forward primer 5'-TTTTTTGAGTTAGGTGTGGG-3' and reverse biotinylated primer 5'-TCTCACTAAAAAATACCAAACAA-3' in a 50 μL reaction volume containing 2.5 ng of bisulfite-treated DNA, 1× PCR buffer, 1.5 mM MgCl_2 _and 1.25 U of Platinum Taq DNA polymerase (Invitrogen, Milan, Italy). PCR thermal amplification profile consisted of an initial denaturation step of 5 min at 95°C, followed by 50 cycles of 30 s at 95°C, 30 s at 58°C, and 1 min at 72°C. The PCR product was purified using Streptavidin Sepharose High Performance beads (Amersham Biosciences, Uppsala, Sweden) and denatured using 0.2 mol/L of NaOH solution. Next, 0.3 μmol/L of the sequencing primer (5'-GGGTGGGAGTGAT-3') was annealed to the purified single-stranded PCR product and the Pyrosequencing reaction was performed using the PSQ HS 96 Pyrosequencing System (Pyrosequencing, Inc., Westborough, MA). The level of methylation for each of the 3 analyzed CpG sites (CpG1, CpG2, CpG3) was expressed as the percentage of methylated cytosines over the sum of methylated and unmethylated cytosines (Figure 1). Within- and between-run variations for the determination of *LINE-1 *methylation through the pyrosequencing assay utilized have been previously described [14].

### Quantitative RT-PCR analysis of LINE-1 mRNA expression

Real-time quantitative RT-PCR analyses were performed essentially as described [15]. Briefly, total RNA was digested with RNAse-free DNAse (Roche Diagnostics, Milan, Italy) to remove contaminating genomic DNA. Synthesis of cDNA was performed on 1 μg total RNA using MMLV reverse transcriptase (Invitrogen, Milan, Italy) and random hexamer primers (Promega, Milan, Italy), following manufacturers' instructions. Real-time quantitative RT-PCR reactions were conducted on the ABI prism 7000 Sequence Detection System (Applied Biosystems, Milan, Italy), utilizing 20 ng retrotranscribed total RNA in a final volume of 25 μl 1 X SYBR Green Master Mix (Applied Biosystems). Relative quantification of LINE-1 mRNA was performed with the aid of the DataAssist v2.0 software (Applied Biosystems), using the β-actin house-keeping gene as endogenous control and normal human PBMC as calibrator. The primers utilized for measurement of LINE-1 (forward, GGCCAGTGTGTGTGCGCACCG; reverse, CCAGGTGTGGGATATAGTCTCGTGG) and of β-actin (forward, CGAGCGCGGCTACAGCTT; reverse, CCTTAATGTCACGCACGATT) mRNA expression were described previously [15,16].

### Statistical analysis

The primary objective was to determine differences in survival among various *LINE-1 *DNA methylation level groups. In order to increase statistical power, sample has been divided in two groups of the same size using median as threshold: CpG1 (<25.68, ≥25.68), CpG2 (<27.26, ≥27.26), and CpG3 (<40.46, ≥40.46). For simplicity groups have been defined as *LINE-1 *hypomethylated (patients with a *LINE-1 *methylation <median) and hyper-methylated (patients with a *LINE-1 *methylation ≥median). The characteristics including age, gender, primary tumor localization, Breslow thickness, Clark level, and ulceration of the primary tumor, number of lymph nodes involved, and pre-operative serum LDH values were examined. Survival time was calculated in months from the date of stage IIIC diagnosis until the date of death. According with the specific goals of the analysis, we did not classify the deaths considering their cause. Patients were censored at the last follow-up date or the last date the patient was last known to be alive. Median survival duration was determined by the Kaplan-Meier method [17]. Cumulative survival by DNA methylation level was evaluated using the log-rank test. P values were two sided and values <0.05 were considered to be statistically significant. Cox proportional hazard method [18] was used to examine the effect of DNA methylation level on survival and results were presented as Hazard Ratios (HR) with corresponding 95% Confidence Intervals (CI). *LINE-1 *methylation was also entered in the model as a continuous variable with the unit set at 10% of methylation. A stepwise regression (forward selection) was conducted to select variables to add in our models. Correlation between *LINE-1 *methylation and mRNA expression was evaluated by Spearman's rank correlation. The statistical analyses were carried out using the SAS Software version 9.13 (SAS Institute Inc., Cary, North Carolina, USA).

## Results

### Patients

The study was conducted on CM patients who underwent radical lymph node dissection for stage III disease at the Centro di Riferimento Oncologico National Cancer Institute between 1991 and 2007. Patients diagnosed with a stage IIIC disease, and for whom a short-term cell culture had been successfully generated from the surgically removed autologous neoplastic tissue, were included in the study. Table 1 summarizes the 42 patients under study and their clinico-pathologic characteristics at presentation.

**Table 1:** Characteristics of the 42 AJCC stage IIIC melanoma patients

Variable	n. patients	%
Age, years		
Median	54	
Range	29-83	
Gender		
Male	27	64
Female	15	36
Localization of primary tumor		
extremities	14	33
trunk	23	55
head & neck	3	7
NA*	2	5
Breslow thickness of primary tumor		
≤2.0 mm	13	31
>2.0 mm	22	52
NA	7	17
Clark level of primary tumor		
1- 3	12	29
4-5	24	57
NA	6	14
Ulceration of primary tumor		
No	10	24
Yes	30	71
NA	2	5
N. lymph nodes involved		
1	9	21
>1	33	79
LDH		
Low_†_	28	67
High	11	26
NA	3	7

*NA, not available

^† ^low LDH is established as LDH values ≤ 0.8 times the upper limit of normal; high LDH is defined as LDH values > 0.8 times the upper limit of normal

### Extent of LINE-1 methylation in CM patients

**Figure 2:**
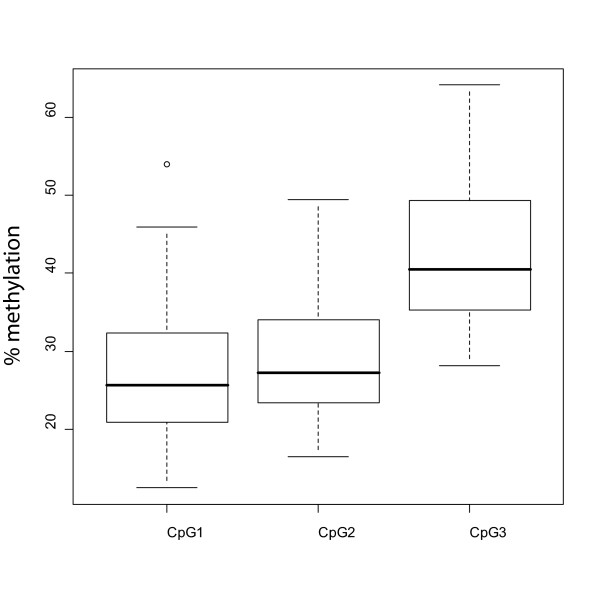
***LINE-1 *methylation in stage IIIC CM patients**. *LINE-1 *methylation at 3 CpG sites (CpG1, CpG2, CpG3) was evaluated by bisulfite pyrosequencing analysis in short-term cultures of CM cells generated from neoplastic lesions of 42 stage IIIC melanoma patients. All cells were analyzed at 6^th ^*in vitro *passage. Separate box plots have been generated for each of the CpG sites under analysis. Black horizontal bars represent the median values of methylation for each group.

To define if CM undergoes changes in the overall content of 5-methylcytosine, bisulfite pyrosequencing analyses (Figure 1) were utilized to measure the extent of methylation of *LINE-1 *repetitive elements in the 42 short-term CM cell cultures under study. Data obtained identified largely heterogeneous levels of methylation of the *LINE-1 *elements in CM cells from stage IIIC patients (CpG1: median 25.68%, range 12.45%-54.05%; CpG2: median 27.26%, range 16.50%-49.43%; CpG3: median 40.46%, range 28.10%-64.15%; Figure 2), demonstrating that highly variable alterations in the overall level of genomic DNA occur in CM. In contrast, homogeneous and high levels of methylation at each of the investigated CpG sites were measured in normal human melanocytes (CpG1: median 62.82%, range 60.43%-67.53%; CpG2: median 52.57%, range 51.37%-52.87%; CpG3: median 65.77%, range 62.40%-67.33%) and in PBMC isolated from healthy donors (CpG1: median 78.0%, range 67.8%-84.2%; CpG2: median 54.7%, range 51.4%-56.8%; CpG3: median 67.9%, range 66.2%-73.3%).

### Prognostic value of LINE-1 methylation in CM patients

The highly heterogeneous levels of *LINE-1 *methylation observed in CM cells from stage IIIC patients led us to investigate whether they correlated with a different clinical outcome of patients under study.

**Figure 3:**
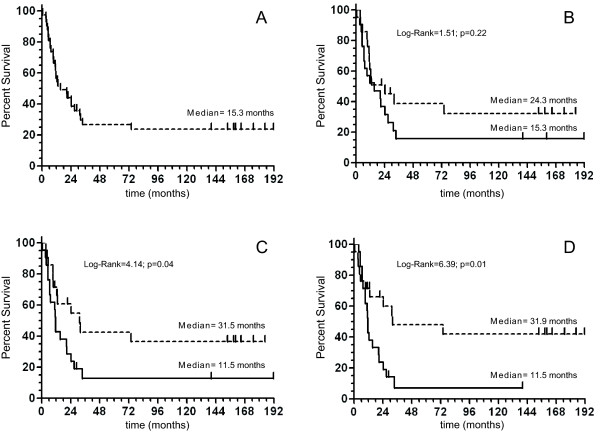
**Kaplan-Meier analysis of CM patients survival according to *LINE-1 *methylation**. *LINE-1 *methylation at 3 CpG sites (CpG1, CpG2, CpG3) was evaluated by bisulfite pyrosequencing analysis in short-term cultures of CM cells generated from neoplastic lesions of 42 stage IIIC melanoma patients. Cells were analyzed at 6^th ^*in vitro *passage. Kaplan- Meyer function for OS was calculated for CM patients either unstratified (A) or stratified according to median methylation of CpG1 (B), CpG2 (C) or CpG3 (D) site of *LINE-1 *elements. Dashed and solid lines refer to patients with *LINE-1 *methylation below or above the median, respectively. Vertical bars represent censored patients. Cumulative survival by *LINE-1 *methylation level was evaluated using the Log-Rank test, reported *P *values were two sided.

Kaplan-Meier analysis indicated that median OS for stage IIIC CM patients under analysis was 15.3 months (95% CI, 11.0-31.5; Figure 3). To evaluate the association between *LINE-1 *methylation status and OS, patients were divided according to the median value of methylation of each analyzed CpG site (CpG1 = 25.68%; CpG2 = 27.26%; CpG3 = 40.46%). Patients were defined as having hypomethylated or hypermethylated *LINE-1 *sequences, depending on the methylation level being below or above the median value for each group, respectively. Kaplan-Meier analysis showed a trend toward an increased OS rate for patients with hypomethylated CpG1, however, the difference did not reach statistical significance (P = 0.22, log-rank = 1.51; Figure 3). On the other hand, a significant survival advantage was observed in patients with CpG2 < 27.26% as compared to patients with CpG2≥27.26% (P = 0.04, log-rank = 4.14) (Figure 3). Similarly, the survival rate of patients with CpG3 < 40.46% was significantly higher than that of patients with CpG3≥40.46% (P = 0.01, log-rank = 6.39) (Figure 3). In line with these data, median OS of patients with hypomethylated CpG1, CpG2 and CpG3 sites was 24.3, 31.5, and 31.9 months, respectively, as compared to 15.3, 11.5, and 11.5 months of patients with hypermethylated *LINE-1 *CpGs (Figure 3, Table 2). Accordingly, the 5 year OS was 39%, 43%, and 48% for patients with hypomethylated CpG1, CpG2, and CpG3 sites, respectively, as compared to 16%, 13%, and 7% of patients with hypermethylated *LINE-1 *CpGs (Table 2).

**Table 2:** OS of stage IIIC CM patients according to *LINE-1 *methylation

LINE1 CpG site	# events/# patients*	**Extent methylation**^**†**^	**Median OS (95%CI)**^**‡**^	5 year OS (%)
**CpG1**	13/21	<25.68	24.3 (11.1-inf)	39
	17/21	≥25.68	15.3 (6.8-26.9)	16
**CpG2**	12/21	<27.26	31.5 (12.5-inf)	43
	18/21	≥27.26	11.5 (6.8-20.9)	13
**CpG3**	11/21	<40.46	31.9 (13.1-inf)	48
	19/21	≥40.46	11.5 (9.2-20.6)	7

* number of patients who died (# events) and total number of patients in the group (# patients) are reported;

† Patients were divided according to the % of methylation of the specified CpG site being < or ≥ the median % methylation measured in the examined patients' population;

^‡ ^Survival functions were calculated by the Kaplan-Meier method. Data are reported as Median OS in months, together with the corresponding 95% Confidence Intervals (CI).

Cox univariate analysis was carried out to identify patient characteristics and clinico-pathologic factors that predicted survival. Among all factors examined, including age, gender, localization of primary tumor, Breslow thickness, Clark level and ulceration of primary tumor, number of lymph nodes involved, and level of pre-operative LDH, only CpG2 methylation (HR = 2.12 for CpG2≥27.26% vs. CpG2 < 27.26; 95% CI: 1.01-4.44; P = 0.04) and CpG3 methylation (HR = 2.63 for CpG2≥40.46% vs. CpG2 < 40.46; 95% CI: 1.21-5.69; P = 0.01) were associated with statistically significant differences in OS (Table 3). A stepwise regression (forward selection) did not point to any independent variable to add in our models, thus, only unadjusted HRs are reported in tables. When *LINE-1 *methylation was analyzed as a continuous variable, a statistically significant inverse association emerged between OS and an increase of 10% of methylation of CpG1 (HR = 1.51; 95%CI:1.06-2.16; P = 0.02), CpG2 (HR = 1.60; 95%CI:1.02-2.52; P = 0.04) and CpG3 (HR = 1.49; 95%CI:1.06-2.09; P = 0.02) (Table 3). The above reported statistically significant increased risk of death associated with *LINE-1 *hypermethylation suggests a potential robust association between methylation at CpG2 and CpG3 and OS, even if the power of our analyses is below 25%.

**Table 3:** Cox analysis of the influence of *LINE-1 *methylation on OS of stage IIIC CM patients

LINE1 CpG site	# events/# patients*	**Extent methylation**^**†**^	**HR**^**‡**^	95% CI; *P *value	**HR**_**cont.**_^**§**^	95% CI; *P *value
**CpG1**	13/21	<25.68	1**		1.51	1.06-2.16; 0.02
	17/21	≥25.68	1.57	0.76-3.24; 0.22		
**CpG2**	12/21	<27.26	1		1.60	1.02-2.52; 0.04
	18/21	≥27.26	2.12	1.01-4.44; 0.04		
**CpG3**	11/21	<40.46	1		1.49	1.06-2.09; 0.02
	19/21	≥40.46	2.63	1.21-5.69;. 0.01		

* number of patients who died (# events) and total number of patients in the group (# patients) are reported;

† Patients were divided according to the % of methylation of the specified CpG site being <or ≥ the median % methylation measured in the examined patients' population;

^‡ ^Cox proportional hazard method was used to examine the effect of *LINE-1 *methylation on OS. Results were presented as Hazard Ratios (HR) with corresponding 95% Confidence Intervals (CI);

^§ ^LINE-1 methylation was also evaluated as continuous variable. The HR value is that of the *LINE-1 *methylation relative to an increase of 10%;

** set as reference.

### Expression of LINE-1 mRNA in CM patients

**Figure 4:**
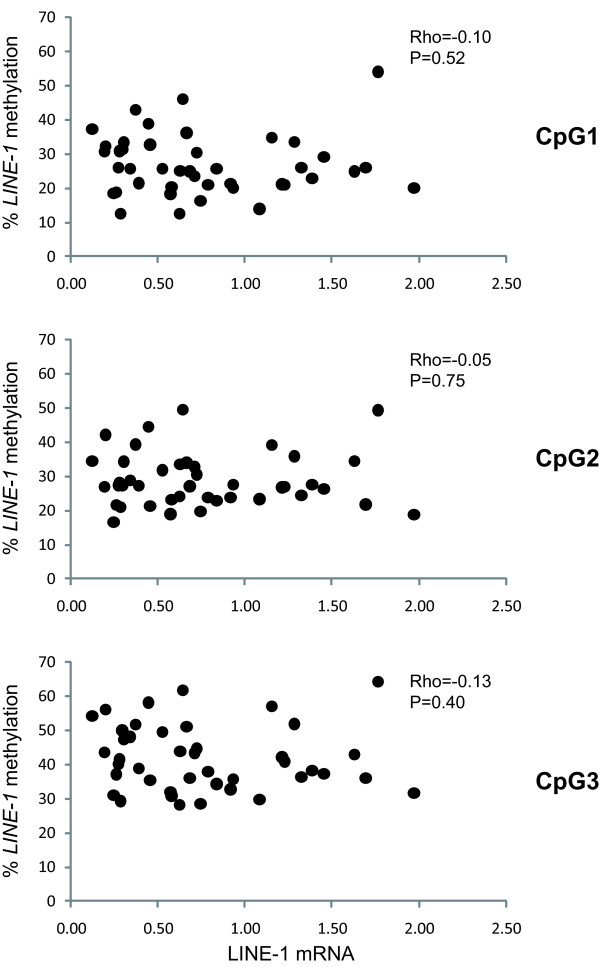
**Association between methylation and mRNA expression of *LINE-1 *elements in stage IIIC CM patients**. Short-term cultures of CM cells generated from neoplastic lesions of 42 stage IIIC melanoma patients were evaluated for *LINE-1 *methylation at 3 CpG sites (CpG1, CpG2, CpG3) and for LINE-1 mRNA expression by bisulfite pyrosequencing and quantitative RT-PCR analyses, respectively. All cells were analyzed at 6^th ^*in vitro *passage. Methylation at each investigated *LINE-1 *CpG site is reported as %, level of LINE-1 mRNA expression is reported relative to the value measured in PBMC obtained from healthy donors, used as reference. Correlation between *LINE-1 *methylation and mRNA expression was evaluated by Spearman's rank correlation test, reported *P *values were two sided.

To provide an initial evaluation of whether the products encoded by the *LINE-1 *repetitive elements might have a direct role in determining the different survival of CM patients with neoplastic cells having different *LINE-1 *methylation statuses, quantitative RT-PCR analyses were utilized to measure the level of LINE-1 mRNA in the 42 short-term CM cell cultures under study. Data obtained revealed heterogeneous levels of LINE-1 mRNA in the CM cell cultures from stage IIIC CM patients (median 0.65, range 0.12-1.97); however, no significant correlation was observed between levels of expression of LINE-1 transcripts and methylation at either CpG1, CpG2 or CpG3 sites (Figure 4).

## Discussion

In this study we demonstrate that the global level of *LINE-1 *methylation of short-term tumor cell cultures grown from patients with nodal disease is a significant predictor of OS in stage IIIC CM patients. This finding is of remarkable clinical relevance, since, to the best of our knowledge, it provides the first evidence of a molecular marker capable of differentiating the prognosis of CM patients in this high-risk substage. These results are of particular emphasis given the conduct of this study in subjects within a single clinically well-defined clinico-pathological staging sub-group, which has become the focus of several ongoing clinical trials in the US and Europe (i.e., ECOG intergroup trial E4697, EORTC trial 18071, GSK trial 111482 "DERMA").

Genomic DNA hypomethylation has been proposed to have an important impact on tumor biology through the generation of chromosomal instability, reactivation of transposable elements, and loss of imprinting [5]. Thus, a negative correlation between genomic hypomethylation and survival of CM patients could have been expected. Instead, we found that hypomethylation of *LINE-1 *elements at CpG2 or CpG3 sites was associated with a significantly better OS, as demonstrated by Kaplan-Meier analysis and log-rank test. The positive prognostic value of *LINE-1 *hypomethylation we have identified in CM is in sharp contrast with data most recently obtained in colon and ovarian cancer patients, in which *LINE-1 *hypomethylation in neoplastic tissues was associated with a poorer prognosis [10,11]. This discrepancy, however, is not completely surprising. Indeed, data generated on hematologic malignancies showed that *LINE-1 *hypomethylation can be either a poor or a good prognostic factor, depending on the patient being affected by chronic myeloid leukemia or acute lymphoblastic leukemia, respectively [19,20]. Thus, the different behavior of CM, with respect to the other solid tumors so far investigated, might further suggest that the underlying biological effect(s) of *LINE-1 *hypomethylation on patients' outcome could depend on the tumor histotype. Nevertheless, it should be emphasized that our findings are generated from patients in the same clinico-pathological stage of disease, while the studies on ovarian and colon cancer were conducted on the heterogeneous patients population as a whole, and did not investigate the prognostic potential of *LINE-1 *methylation in specific clinically defined stages of disease. Thus, it remains to be demonstrated whether this different study approach might contribute to the observed discrepancy. Furthermore, it cannot be ruled out that in the different sources of neoplastic material analyzed, the presence of varying proportions of contaminating normal cells in neoplastic tissues, as well as the different methodological approaches employed might contribute to conclusions that may differ from those we have reached in these studies. In this context, our use of short-term CM cultures has the advantage of eliminating contaminating normal cells, yet representing the methylation status of neoplastic cells of the fresh autologous lesion. In fact, similar levels of *LINE-1 *methylation were observed between short-term cultures and autologous uncultured CM cells that were purified by anti-HMW-MAA immunomagnetic beads from tumor cell suspensions that were available from 10 patients (data not shown).

The mechanism(s) through which *LINE-1 *hypomethylation affects survival of CM patients remains to be fully explored; however, some speculation can be made, based on recent data in the literature. Tellez *et al *[21] have demonstrated that higher levels of *LINE-1 *methylation correlate with an increased number of aberrantly hypermethylated tumor suppressor genes (TSG) in cultured melanoma cell lines. This notion has gained further support from our most recent observation showing a direct correlation between higher *LINE-1 *methylation and increased genome-wide gene methylation, measured through CpG island microarrays (Sigalotti and Maio, manuscript in preparation). Thus, epigenetic inactivation of TSG might account for more aggressive disease we have observed in patients with elevated *LINE-1 *methylation in their neoplastic cells. This hypothesis is in accordance with initial studies reporting a negative association between survival and the presence of hypermethylated *ER-α*, *RASSF1A*, *RAR-β2, or MINT31 *DNA in neoplastic tissues or sera of stage III/IV CM patients [22-24]. On the other hand, hypomethylation, and consequent transcriptional activation, of *LINE-1 *elements might *per se *reduce the tumorigenic potential of neoplastic cells by triggering apoptosis and a senescence-like state through the activity of the second open reading frame of *LINE-1 *[25]. In our findings, this seems not to be the case, since the lack of correlation between methylation and mRNA expression of *LINE-1 *elements, suggests that *LINE-1 *products may not be the driving force for the observed increased OS of *LINE-1 *hypomethylated patients. Genomic DNA hypomethylation has also been associated with the *de novo *expression of tumor associated antigens belonging to the Cancer Testis Antigen (CTA) class by neoplastic cells of different histotype, including melanoma stem cells [26-29], and we have recently identified a significant correlation between a hypomethylated status of *LINE-1 *elements and increased levels and total number of CTA concomitantly expressed in short-term cultures of CM cells (Sigalotti and Maio, unpublished). Besides, pharmacologic DNA hypomethylation has been consistently demonstrated to increase immunogenicity and immune recognition of cancer cells through the up-regulation of different molecules involved in antigen processing and presentation, including HLA class I antigens and co-stimulatory molecules [30,31]. Thus, it is intriguing to speculate that a better immune recognition of *LINE-1 *hypomethylated CM cells might contribute to the improved survival of these patients. This hypothesis may find indirect support from most recent gene expression profiling studies that identified the expression of "immune-related" genes in the tumor as a marker of good prognosis in stage III-IV CM [32-34].

## Conclusion

Irrespective of the underlying biological mechanism(s) triggered by *LINE-1 *hypomethylation, the prognostic value of *LINE-1 *methylation here identified for stage IIIC CM patients bears several important practical clinical implications. Among these, the goal to provide CM patients with improved clinico-pathological sub-stage and/or follow-up-procedures would be enhanced using *LINE-1 *methylation status, and these findings might be used to select and/or stratify patients for adjuvant treatment based on the methylation level of *LINE-1 *in their tumors. In addition, the significant positive prognosis of *LINE-1 *hypomethylated patients should prompt the incorporation of this in new studies aimed at understanding whether pharmacologic DNA hypomethylation [35] could be regarded as a feasible chemoprevention approach in the initial phases of disease and/or in patients at high-risk of disease recurrence.

Our present findings will be further investigated in prospective multicenter studies in which the prognostic significance and the predictive value for different treatments of CM will be validated. Providing further support to our initial data will finally allow to establish the appropriateness of adding the evaluation of *LINE-1 *methylation into the routine clinico-pathological ascertainment of CM patients, in order to help personalizing their comprehensive clinical management.

## List of Abbreviations Used

CI: Confidence Intervals; CM: cutaneous melanoma; CTA: Cancer Testis Antigen; ER-α: Estrogen Receptor-α; HLA: Human Leukocyte Antigen; HMW-MAA: High Molecular Weight-Melanoma Associated Antigen; HR: Hazard Ratio; LINE-1: Long Interspersed Nucleotide Element-1; MINT31: Methylated IN Tumors locus 31; OS: overall survival; RASSF1A: Ras Association (RalGDS/AF-6) domain Family member 1A; RAR-β2: Retinoic Acid Receptor-β2; TSG: tumor suppressor genes.

## Competing interests

LS and MM have applied for a patent based on the findings reported in this manuscript. All other authors declare no competing interests.

## Authors' contributions

LS participated in acquiring laboratory data, data analysis and interpretation, study coordination, and drafted the manuscript. EF performed the pyrosequencing analyses, and contributed in data acquisition and analysis. EB performed the statistical analyses. AC, GP, FC contributed in cellular biology procedures, molecular assays and data acquisition. SC, contributed in data interpretation. SM participated in acquisition of clinical data and data interpretation. JMK participated in data interpretation and manuscript drafting. MM conceived of the study, participated in its design and coordination, and contributed in producing the final draft of the manuscript. All authors read and approved the final manuscript.
